# The Carcinoma Mimic: A Case of Breast Fibromatosis

**DOI:** 10.7759/cureus.98431

**Published:** 2025-12-04

**Authors:** Allison Draper, Madeleine Hotaling, Elise Hotaling

**Affiliations:** 1 General Surgery, Broward Health, Fort Lauderdale, USA; 2 Jupiter Environmental Research and Field Studies Academy, Jupiter High School, Jupiter, USA; 3 Breast Imaging, Virtual Radiologic, Jupiter, USA

**Keywords:** breast cancer, breast fibromatosis, breast surgical oncology, desmoid tumor, diagnostic radiology, diagnostic radiology education

## Abstract

Fibromatosis of the breast is a rare and locally aggressive disease that mimics carcinoma on imaging. We present a case of a 17-year-old female who presented with a painless, palpable 8 cm lump in her left breast. Breast ultrasound highlighted features concerning for malignancy - a large, irregular, hypoechoic mass with posterior acoustic shadowing. However, the absence of a hyperechoic halo, presence of posterior acoustic attenuation, and low vascularity pointed towards the correct diagnosis. In addition, on magnetic resonance imaging (MRI), breast fibromatoses correspond to hyperintense signals on T2, which, though a nonspecific finding, may be seen with myxoid tissue and hypointense signals on T1. Following contrast administration, there was progressive enhancement as opposed to the typical washout kinetics of breast carcinoma. Diagnosis of breast fibromatosis was confirmed on core needle biopsy. Although the differential for an irregular hypoechoic breast mass should be broad, it can be narrowed with history and radiographic findings. This case report highlights the unique ultrasound and MRI findings seen in breast fibromatoses to spread awareness of active surveillance and improve early detection.

## Introduction

Fibromatosis of the breast is a rare, benign, stromal tumor characterized by fibroblastic proliferation with infiltrative growth. These lesions account for less than 0.2% of all breast tumors and can occur secondary to prior trauma or may be linked to mutations resulting in dysregulation of β-catenin [1-2]. Although histologically nonmalignant, these lesions are locally aggressive and have a strong tendency for recurrence following surgical excision. Breast fibromatoses most commonly present in women between their fourth and fifth decades of life, and can provide quite a diagnostic dilemma.

Clinically and radiographically, breast fibromatosis frequently mimics invasive carcinoma, presenting as a firm, irregular mass with indistinct margins on imaging [2]. Because of this overlap, patients may undergo extensive diagnostic workup or surgical excision before a definitive diagnosis is made. This workup exposes the patient to unnecessary risk, especially for a benign condition that will often go unchanged or even spontaneously resolve. Recognition of characteristic imaging findings and correlation with patient history are key to narrowing the differential before tissue confirmation. We present a case of breast fibromatosis in a 17-year-old female, highlighting the diagnostic considerations and imaging features that help distinguish this entity from malignancy.

## Case presentation

A 17-year-old female presented in January of 2024 with a painless, palpable lump in the upper-outer quadrant of her left breast discovered on self-exam. The patient had no prior trauma or medical problems, no family history of breast or ovarian cancer, and no prior pregnancies. She had regular menstrual cycles and had never been on birth control.

The patient was evaluated by a breast surgical oncologist. During this exam, she was found to be afebrile, hemodynamically stable, and saturating well on room air. On physical exam, there was a large, nonmobile, hard mass in the left upper quadrant of the left breast without overlying skin changes. No other breast masses or axillary lymphadenopathy were appreciated. The patient underwent a breast ultrasound that showed an abnormality that was further investigated with magnetic resonance imaging (MRI). 

**Figure 1:**
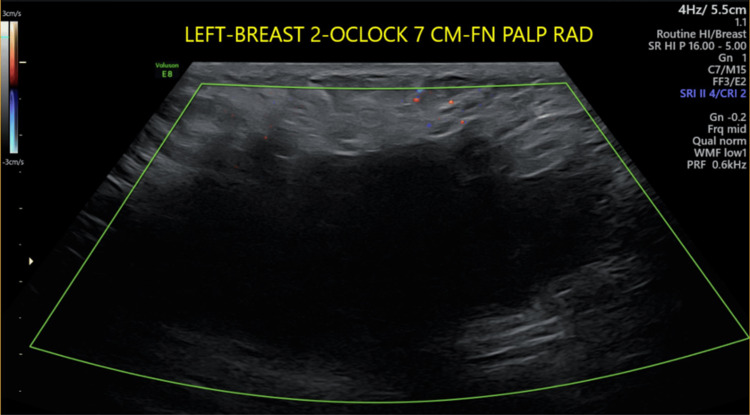
Color Doppler ultrasound of the breast Color Doppler ultrasound image showing no vascular flow to the left breast irregular hypoechoic mass with angular margins and posterior acoustic shadowing.

Ultrasound of the palpable lump in the upper-outer quadrant of the left breast demonstrated an 8.1x3.0x5.9 cm irregular hypoechoic mass with angular margins and posterior acoustic shadowing (Figure 1). The posterior margin of the mass was difficult to distinguish from the adjacent musculature. No surrounding hyperechoic halo was present, and color Doppler imaging demonstrated no internal vascularity.

**Figure 2:**
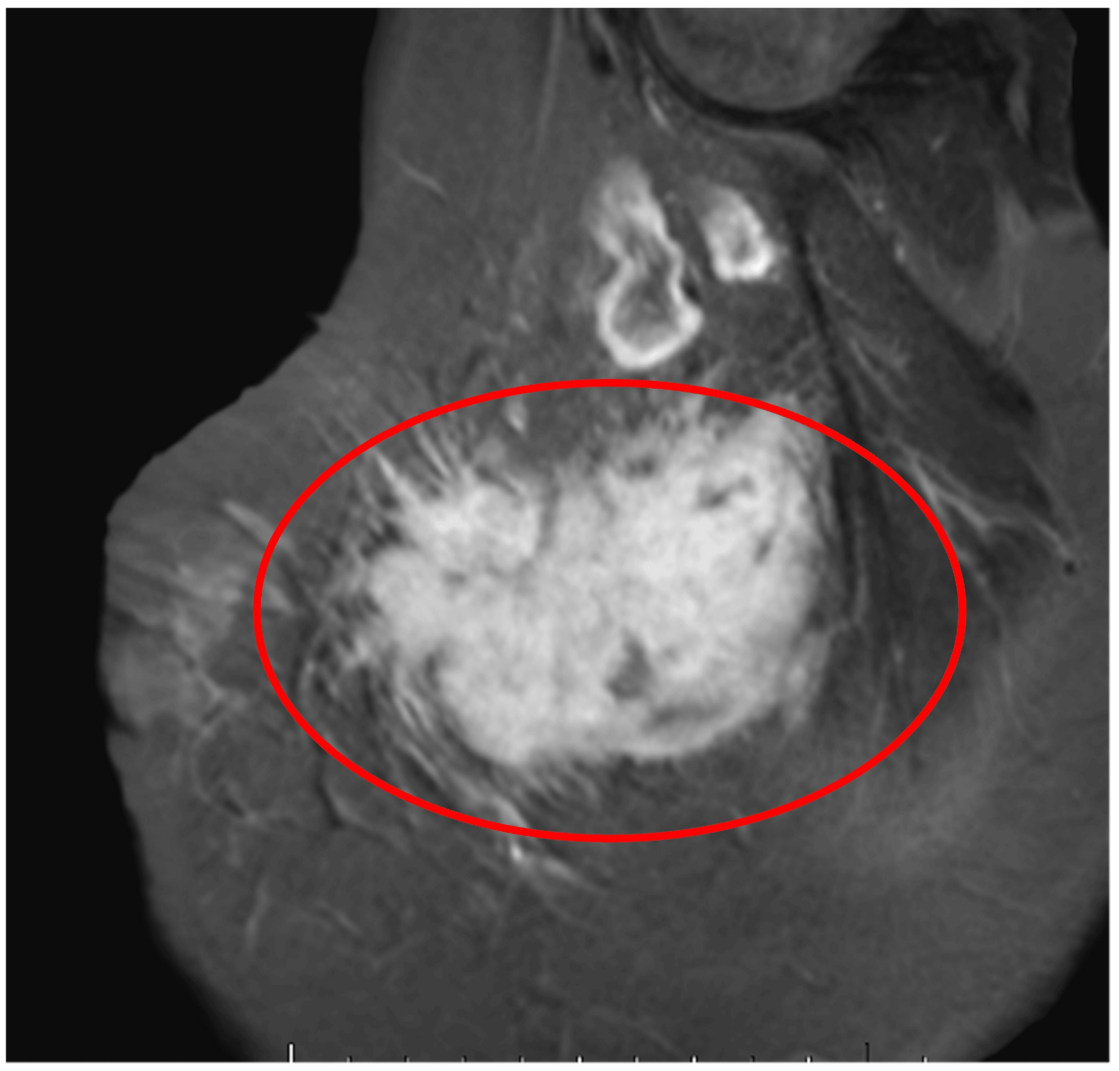
Sagittal T2-weighted STIR scan of the left breast Sagittal T2-weighted STIR scan of the left breast (repetition time scan/echo time msec, 3.5/57, section thickness 5mm) showed the mass to be hyperintense. STIR: Short Tau Inversion Recovery

**Figure 3:**
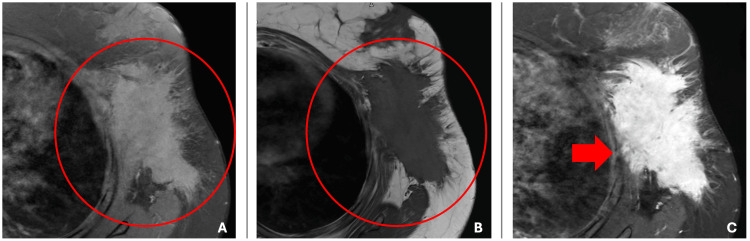
Axial contrast enhanced MRI scans of the left breast on 3T (A) Axial T1-weighted imaging showed the mass to be hypointense (repetition time scan/echo time msec, 636/10, section thickness 6 mm). Axial T1-weighted turbo spin echo (B) without fat saturation (repetition time scan/echo time msec, 636/10, section thickness 6 mm) and (C) with fat saturation (repetition time scan/echo time msec, 640/10, section thickness 6 mm) showed the mass to be within the breast with the involvement of the adjacent pectoralis, anterior serratus, and latissimus dorsi musculature, and no distinguishable intervening fat plane showing fascial involvement (red arrow).

**Figure 4:**
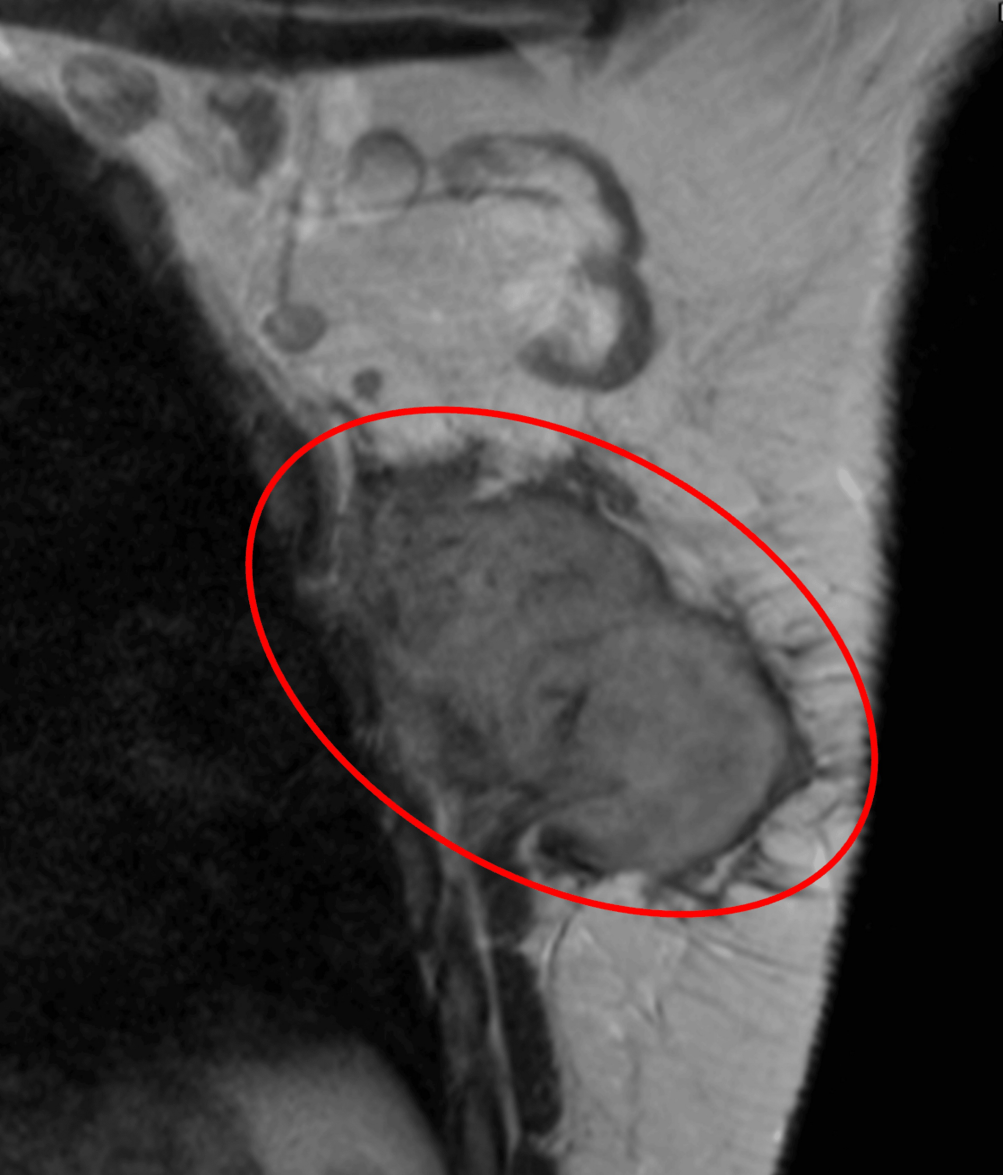
Coronal contrast-enhanced MRI scan of the left breast acquired on 3T Coronal contrast-enhanced MRI scan of the left breast acquired on 3T (9mL gadobutrol) T2-weighted Dixon image (repetition time scan/echo time msec, 3.840/94, section thickness 5 mm) showed heterogeneous progressive internal enhancement of the mass with irregular margins.

The mass was further characterized with MRI, which demonstrated a large spiculated mass in the upper-outer quadrant of the left breast, correlating with the palpable lump and the mass seen on the ultrasound. On axial T1-weighted image, the mass was hypointense (Figure 2A), and on sagittal T2-weighted image, the mass was hyperintense (Figure 3). The axial T1-weighted turbo spin echo with and without fat saturation demonstrated that the mass tented the adjacent pectoralis, anterior serratus, and latissimus dorsi musculature. With no distinguishable intervening fat plane, it was difficult to determine whether the mass arose in the breast and invaded the adjacent muscles or if the mass arose in the musculature and invaded the adjacent breast tissue (Figure 2B, 2C). Coronal contrast-enhanced T2-weighted image showed heterogeneous progressive internal enhancement of the mass with irregular margins (Figure 4). The adjacent lymph nodes were within normal limits.

The diagnosis of fibromatosis was confirmed with core needle biopsy. A decision was made to forgo surgical excision and manage the patient with active surveillance. Her most recent MRI in February of 2025 demonstrated stable disease without interval change.

## Discussion

The age of this patient should prompt the first hesitation in diagnosis. While not impossible, breast carcinoma in a 17-year-old woman is highly improbable, especially in one with no family history or known risk factors. Fibromatoses are either secondary to prior trauma or linked to mutations resulting in dysregulation of β-catenin, such as mutations in the adenomatous polyposis coli (*APC*) gene [1]. Most cases present in reproductive-aged women, with some cases documented in patients as young as their early 20s [2]. 

The early age of presentation in our case necessitates a careful radiologic evaluation to broaden the differential. While it is important to include carcinoma in the differential given the irregular, hypoechoic mass, specific clues point towards the correct diagnosis. Characteristics that can independently differentiate breast fibromatosis from invasive ductal carcinoma on ultrasound include the absence of a hyperechoic halo, presence of posterior acoustic attenuation, and low vascularity [3]. A peripheral hyperechoic halo is the result of lymphatic infiltration and subsequent edema often found in infiltrating carcinoma - a finding less likely to be seen in a benign process. Posterior acoustic shadowing is an ultrasound finding seen when the sound wave is absorbed or reflected by the tissue, often indicating solid tissue. The lack of vascularity indicates it may more likely reflect fibrotic tissue rather than a neoplasm. Fibromatoses are composed of fibroblasts with considerable extracellular collagen, contributing to the posterior echo attenuation and absence of vascularity. 

Our case was further evaluated with MRIs, of which the kinetic evaluation provides the greatest diagnostic utility. Breast fibromatoses correspond to hyperintense signals on T2 and hypointense signals on T1 with progressive enhancement as opposed to the typical washout kinetics of breast carcinoma [4-6]. In our patient, MRI showed a heterogeneous spiculated mass with involvement of the chest wall musculature (Figure 4). The mass was hypointense on T1 with persistent internal enhancement and hyperintense on T2-weighted imaging (Figure 2A, Figure 3). This gradual enhancement is proposed to be, in part, due to the collagenous component and myxoid changes of the tumor, and contrasts with the rapid washout seen in invasive carcinoma. In addition, the involvement of the mass with the chest wall helps to point toward fibromatosis, as these lesions are typically associated with muscle or fascia (Figure 2B, 2C). The increased signal intensity on T2 is secondary to the dense collagenous tissue and increased cellularity as fibroblasts and myofibroblasts are typically iso or hypointense to skeletal muscle. While a hyperintense T2 signal is typically seen in fibromatoses, it is a nonspecific finding. Detection rates are higher when lesions involve the fascial planes, as fascial extension is easily differentiated from the surrounding fat [7].

While our patient did not undergo a mammogram, if she had, it would not have provided a greater diagnostic utility than ultrasound and MRI. Fibromatoses have a similar density to glandular tissue, making differentiation difficult. Because of this, abnormality detection rates on mammography vary greatly, ranging between 33% [7], 79% [2], and 92% [8]. Additionally, if the mass arises from the pectoralis muscle, the mass may not be in the field of view on mammography. Mammographic imaging findings are non-specific and include irregular masses with architectural distortion and spiculation, skin dimpling from retraction thickening, or nipple retraction. 

The diagnosis was confirmed with a core needle biopsy showing long sweeping fascicles of bland spindle cells around normal glandular tissue. The cells showed abnormal β-catenin nuclear positivity with the absence of Cluster of Differentiation 34 (CD34) staining, aligning with the pathophysiology of dysregulation of β-catenin in breast fibromatoses [1,9]. Ultimately, our patient was found to carry a pathologic variant in exon 3 of the Catenin Beta-1 (*CTNNB1*) gene (c.121A>G), which codes for the production of β-catenin, providing an explanation for her early presentation of such a rare disease.

The differential for an irregular, hypoechoic breast mass should be broad but can be narrowed with history and radiographic findings. Phyllodes tumors rarely invade the chest wall [10]. Neurofibromas typically present as a well-circumscribed oval mass with posterior enhancement on ultrasound [11]. Nodular fasciitis lesions tend to be subcutaneous, small, painful, and well-circumscribed lesions [12]. Sarcomas are difficult to differentiate from fibromatoses on imaging characteristics alone, as histologic stains are required to make a final diagnosis [13]. However, imaging remains valuable in guiding management after biopsy is performed.

A wide radiologic differential is crucial in these cases, as treatment guidelines have recently changed. Historically, these were treated with wide local excision; however, recurrence after excision (13%), frequent mass stability (52%), and possible spontaneous resolution (36%) prompted a transition to active surveillance [14]. This includes an initial clinical exam and MRI, with repeat imaging every 3-6 months. In smaller, more discrete masses, ultrasound follow-up may be performed to assess changes in size or regression of the mass. Surgical excision or systemic therapy is reserved for progression of disease on active surveillance.

## Conclusions

This rare case of breast fibromatosis in a 17-year-old patient highlights the importance of recognizing the pathology's distinct sonographic and MRI features to avoid unnecessary surgical intervention. On ultrasound, the absence of a hyperechoic halo, posterior acoustic attenuation, and low vascularity helped distinguish the lesion from invasive carcinoma. On MRI, the lesion demonstrated T1 hypointensity, T2 hyperintensity, and gradual enhancement rather than the rapid washout kinetics characteristic of many malignant masses. Increased awareness of this rare entity can support timely diagnosis and guide the appropriate use of active surveillance as a safe and effective management strategy.
